# Bis[μ-*N*,*N*′-bis(2,6-diisopropylphenyl)ethene-1,2-diamido]-1,4(η^2^);1:2κ^4^ *N*:*N*;3:4κ^4^ *N*:*N*-bis(diethyl ether)-1κ*O*,4κ*O*-di-μ-hydrido-2:3κ^4^ *H*:*H*-2,3-dichromium(II)-1,4-dilithium(I) pentane hemisolvate

**DOI:** 10.1107/S160053681000560X

**Published:** 2010-02-13

**Authors:** Stephan Peitz, Normen Peulecke, Bernd H. Müller, Anke Spannenberg, Uwe Rosenthal

**Affiliations:** a Leibniz-Institut für Katalyse e. V. an der Universität Rostock, Albert-Einstein-Str. 29a, 18059 Rostock, Germany

## Abstract

The title compound, [Cr_2_Li_2_(C_26_H_36_N_2_)_2_(μ-H)_2_(C_4_H_10_O)_2_]·0.5C_5_H_12_, is a binuclear chromium complex bridged by two hydrogen atoms. Each chromium atom is coordinated in a distorted square-planar geometry by one chelating bis­(2,6-diisopropyl­phen­yl)ethene-1,2-diamido ligand *via* its two N atoms. Additionally, two diametrically opposed lithium ether adducts coordinate in an η^4^ mode on the backbone of the ligands. There is a crystallographic inversion center in the middle of the Cr_2_H_2_ ring. One of the isopropyl groups is disordered over two positions in a 0.567 (7):0.433 (7) ratio. Disorder is also observed in the pentane hemisolvate molecule.

## Related literature

For other binuclear dihydrido-bridged chromium complexes, see: Fryzuk *et al.* (1994 ▶), MacAdams *et al.* (2003 ▶), Albahily *et al.* (2008 ▶); Rozenel *et al.* (2009 ▶). For the role of binuclear dihydrido-bridged chromium complexes in selective oligomer­ization of ethyl­ene, see: Overett *et al.* (2005 ▶). For similar coordination of alkali metals in a metal–diimine complex, see: Baker *et al.* (2005 ▶). For the binuclear starting compound of this synthesis, see: Peitz *et al.* (2009 ▶).
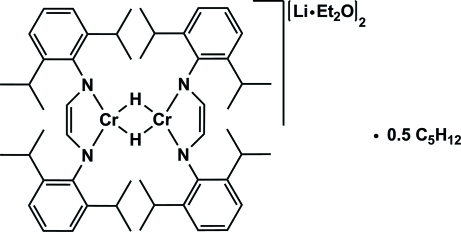


## Experimental

### 

#### Crystal data

[Cr_2_Li_2_(C_26_H_36_N_2_)_2_H_2_(C_4_H_10_O)_2_]·0.5C_5_H_12_
*M* *_r_* = 1057.34
Triclinic, 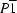

*a* = 12.2577 (5) Å
*b* = 12.3525 (6) Å
*c* = 12.9708 (6) Å
α = 67.827 (4)°
β = 75.039 (3)°
γ = 66.773 (3)°
*V* = 1657.46 (14) Å^3^
*Z* = 1
Mo *K*α radiation
μ = 0.37 mm^−1^
*T* = 200 K
0.50 × 0.40 × 0.35 mm

#### Data collection

STOE IPDS II diffractometer
Absorption correction: numerical (*X-SHAPE* and *X-RED32*; Stoe & Cie, 2005 ▶) *T*_min_ = 0.809, *T*_max_ = 0.905
23828 measured reflections
6500 independent reflections
4770 reflections with *I* > 2σ(*I*)
*R*_int_ = 0.029

#### Refinement

*R*[*F* ^2^ > 2σ(*F* ^2^)] = 0.052
*wR*(*F* ^2^) = 0.151
*S* = 0.99
6500 reflections
337 parameters
53 restraints
H atoms treated by a mixture of independent and constrained refinement
Δρ_max_ = 0.69 e Å^−3^
Δρ_min_ = −0.59 e Å^−3^

### 

Data collection: *X-AREA* (Stoe & Cie, 2005 ▶); cell refinement: *X-AREA*; data reduction: *X-AREA*; program(s) used to solve structure: *SHELXS97* (Sheldrick, 2008 ▶); program(s) used to refine structure: *SHELXL97* (Sheldrick, 2008 ▶); molecular graphics: *XP* in *SHELXTL* (Sheldrick, 2008 ▶); software used to prepare material for publication: *SHELXTL*.

## Supplementary Material

Crystal structure: contains datablocks I, global. DOI: 10.1107/S160053681000560X/im2181sup1.cif

Structure factors: contains datablocks I. DOI: 10.1107/S160053681000560X/im2181Isup2.hkl

Additional supplementary materials:  crystallographic information; 3D view; checkCIF report

## Figures and Tables

**Fig. 1.**
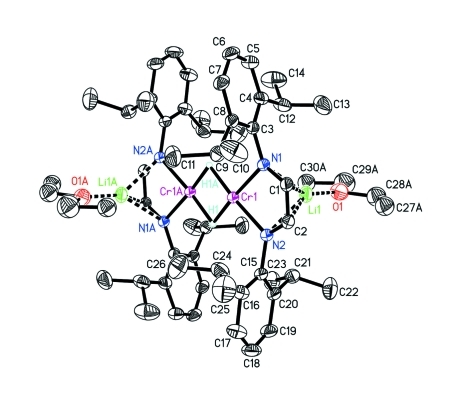
The molecular structure of the title compound showing the atom-labelling scheme. Thermal ellipsoids are drawn at the 30% probability level. Hydrogen atoms, solvent and the disorder of the coordinated diethyl ether are not shown for clarity.

## supplementary crystallographic information

### Comment

Structurally characterized binuclear chromium complexes that are bridged by two hydrogen atoms were reported only four times before (Fryzuk *et al.*, 1994; MacAdams *et al.*, 2003; Albahily *et al.*, 2008; Rozenel *et al.*, 2009). This is the first time such kind of complex is reported with ethene-1,2-diamido ligands. A similar coordination of alkali metals on binuclear metal-1,2-diiminoethane complexes was observed before (Baker *et al.*, 2005).

We became interested in chromium hydride derivatives because they are postulated as intermediates formed in selective oligomerization of ethylene following β-hydride elimination of the hepta- or nonametallacycle, prior to elimination of 1-hexene or 1-octene. Cr dihydride species are suggested to be formed in side-chain reactions during the ethylene tetramerization process (Overett *et al.*, 2005). In order to explore the chemistry of these kinds of complexes, we reacted a binuclear chromium diimine complex (Peitz *et al.*, 2009) with 1,4-dilithiobutane to build up chromacyclopentanes which decompose at room temperature and form a binuclear dihydrido-bridged chromium complex.

The molecular structure of the title compound shows that two chromium(II) centers are bridged by two hydrogen atoms to form a binuclear complex. Each metal center is coordinated by one chelating diimine igand, (*i*-Pr)_2_C_6_H_3_—NC(H)—C(H)N—C_6_H_3_(*i*-Pr)_2_, via both N atoms of each ligand. Due to its redox properties this ligand acts as electron acceptor which leads to the shortened C—C and elongated C—N bond lengths in the ligand backbone in comparison to the free diimine ligand, thus forming an ethene-1,2-diamido unit. Additionally, diametrically opposed to each other, two lithium ether adducts coordinate in a η^4^ mode on the backbone of the ligands which are twisted in an angle of 62.1 (1) ° against each other. The coordination geometry on each chromium center can be best described as distorted square planar (mean deviation from the best plane defined by Cr1—N1—C1—C2—N2 0.046 Å). The Cr1—Cr1' distance of 2.5779 (5) Å is around 0.14 Å shorter than those found in all the other structurally characterized dihydride-bridged chromium dimers and can be interpreted in terms of metal-metal interactions. The Cr—H distances found (both 1.71 (3) Å) are comparable to those of Fryzuk *et al.* (1.78 (3) and 1.76 (3) Å), MacAdams *et al.* (1.77 (3) and 1.77 (3) Å), Rozenel *et al.* (1.84 (2) and 1.85 (2) Å) and Albahily *et al.* (1.69 and 1.68 Å). The asymmetric unit contains one half of the complex unit and a quarter solvent molecule *n*-pentane. The other half of the complex unit and a further quarter solvent molecule are generated by the crystallographic inversion center located in the middle of the Cr_2_H_2_ ring.

### Experimental

1.55 ml of a 0.24 M solution of 1,4-dilithiobutane in diethyl ether were added dropwise to a solution of [(C_26_H_36_N_2_)CrCl(µ-Cl)_3_Cr(THF)(C_26_H_36_N_2_)].CH_2_Cl_2_ (0.40 g, 0.37 mmol) in 2 ml diethyl ether at -78 °C. After stirring over night the solution was filtered and all volatiles were removed in vacuum. Extraction with *n*-pentane gave a green solution. Crystallization at -30 °C yielded 0.082 g (22%) of red-brown single crystals suitable for X-ray analysis.

### Refinement

H1 was located via the difference Fourier map and refined isotropically. All other H atoms were placed in idealized positions with d(C—H) = 0.99 (CH_2_), 0.98 (CH_3_) and 0.95-1.00 Å (CH) and refined using a riding model with *U*_iso_(H) fixed at 1.5 *U*_eq_(C) for CH_3_ and 1.2 *U*_eq_(C) for CH_2_ and CH.

### Crystal data

**Table:**

[Cr_2_Li_2_(C_26_H_36_N_2_)_2_H_2_(C_4_H_10_O)_2_]·0.5C_5_H_12_	*Z* = 1
*M**_r_* = 1057.34	*F*(000) = 573
Triclinic, *P*1	*D*_x_ = 1.059 Mg m^−^^3^
Hall symbol: -P 1	Mo *K*α radiation, λ = 0.71073 Å
*a* = 12.2577 (5) Å	Cell parameters from 19482 reflections
*b* = 12.3525 (6) Å	θ = 1.8–29.1°
*c* = 12.9708 (6) Å	µ = 0.37 mm^−^^1^
α = 67.827 (4)°	*T* = 200 K
β = 75.039 (3)°	Prism, red-brown
γ = 66.773 (3)°	0.50 × 0.40 × 0.35 mm
*V* = 1657.46 (14) Å^3^	

### Data collection

**Table:**

STOE IPDS II diffractometer	6500 independent reflections
Radiation source: fine-focus sealed tube	4770 reflections with *I* > 2σ(*I*)
graphite	*R*_int_ = 0.029
ω scans	θ_max_ = 26.0°, θ_min_ = 1.9°
Absorption correction: numerical (*X-SHAPE* and *X-RED32*; Stoe & Cie, 2005)	*h* = −15→14
*T*_min_ = 0.809, *T*_max_ = 0.905	*k* = −15→15
23828 measured reflections	*l* = −15→15

### Refinement

**Table:**

Refinement on *F*^2^	Primary atom site location: structure-invariant direct methods
Least-squares matrix: full	Secondary atom site location: difference Fourier map
*R*[*F*^2^ > 2σ(*F*^2^)] = 0.052	Hydrogen site location: inferred from neighbouring sites
*wR*(*F*^2^) = 0.151	H atoms treated by a mixture of independent and constrained refinement
*S* = 0.99	*w* = 1/[σ^2^(*F*_o_^2^) + (0.1013*P*)^2^] where *P* = (*F*_o_^2^ + 2*F*_c_^2^)/3
6500 reflections	(Δ/σ)_max_ = 0.001
337 parameters	Δρ_max_ = 0.69 e Å^−^^3^
53 restraints	Δρ_min_ = −0.59 e Å^−^^3^

### Special details

**Table:**

Geometry. All esds (except the esd in the dihedral angle between two l.s. planes) are estimated using the full covariance matrix. The cell esds are taken into account individually in the estimation of esds in distances, angles and torsion angles; correlations between esds in cell parameters are only used when they are defined by crystal symmetry. An approximate (isotropic) treatment of cell esds is used for estimating esds involving l.s. planes.
Refinement. Refinement of *F*^2^ against ALL reflections. The weighted *R*-factor wR and goodness of fit *S* are based on *F*^2^, conventional *R*-factors *R* are based on *F*, with *F* set to zero for negative *F*^2^. The threshold expression of *F*^2^ > σ(*F*^2^) is used only for calculating *R*-factors(gt) etc. and is not relevant to the choice of reflections for refinement. *R*-factors based on *F*^2^ are statistically about twice as large as those based on *F*, and *R*- factors based on ALL data will be even larger.

### Fractional atomic coordinates and isotropic or equivalent isotropic displacement parameters (Å^2^)

**Table:**

	*x*	*y*	*z*	*U*_iso_*/*U*_eq_	Occ. (<1)
C1	−0.0138 (2)	0.3858 (2)	0.8461 (2)	0.0448 (6)	
H1A	−0.0463	0.3863	0.9250	0.054*	
C2	0.0861 (2)	0.2969 (2)	0.8219 (2)	0.0455 (6)	
H2A	0.1321	0.2279	0.8822	0.055*	
C3	−0.1878 (2)	0.5549 (2)	0.7761 (2)	0.0413 (6)	
C4	−0.2201 (2)	0.6843 (3)	0.7494 (2)	0.0486 (6)	
C5	−0.3402 (3)	0.7530 (3)	0.7716 (3)	0.0574 (7)	
H5A	−0.3628	0.8404	0.7527	0.069*	
C6	−0.4265 (3)	0.6977 (3)	0.8201 (3)	0.0580 (7)	
H6A	−0.5072	0.7459	0.8367	0.070*	
C7	−0.3951 (2)	0.5724 (3)	0.8441 (2)	0.0529 (7)	
H7A	−0.4552	0.5345	0.8769	0.063*	
C8	−0.2766 (2)	0.4982 (2)	0.8218 (2)	0.0437 (6)	
C9	−0.2502 (2)	0.3615 (3)	0.8423 (2)	0.0516 (7)	
H9A	−0.1623	0.3246	0.8208	0.062*	
C10	−0.2791 (5)	0.2931 (4)	0.9650 (4)	0.1036 (15)	
H10A	−0.2366	0.3068	1.0109	0.155*	
H10B	−0.2538	0.2043	0.9755	0.155*	
H10C	−0.3655	0.3242	0.9879	0.155*	
C11	−0.3145 (4)	0.3402 (4)	0.7697 (4)	0.0919 (13)	
H11A	−0.2949	0.2510	0.7854	0.138*	
H11B	−0.2890	0.3776	0.6903	0.138*	
H11C	−0.4012	0.3783	0.7865	0.138*	
C12	−0.1263 (3)	0.7472 (3)	0.7032 (3)	0.0647 (8)	
H12A	−0.0619	0.6995	0.6561	0.078*	
C13	−0.0695 (4)	0.7370 (5)	0.7996 (4)	0.0964 (14)	
H13A	−0.0403	0.6499	0.8452	0.145*	
H13B	−0.1293	0.7853	0.8463	0.145*	
H13C	−0.0025	0.7691	0.7692	0.145*	
C14	−0.1712 (4)	0.8802 (4)	0.6282 (4)	0.1036 (16)	
H14A	−0.2060	0.8829	0.5668	0.155*	
H14B	−0.1043	0.9125	0.5971	0.155*	
H14C	−0.2323	0.9310	0.6722	0.155*	
C15	0.2134 (2)	0.2000 (2)	0.6846 (2)	0.0457 (6)	
C16	0.1805 (3)	0.0945 (3)	0.7132 (3)	0.0584 (7)	
C17	0.2667 (3)	−0.0132 (3)	0.6939 (3)	0.0726 (9)	
H17A	0.2457	−0.0856	0.7147	0.087*	
C18	0.3814 (3)	−0.0162 (3)	0.6454 (3)	0.0731 (10)	
H18A	0.4395	−0.0906	0.6344	0.088*	
C19	0.4114 (3)	0.0889 (3)	0.6129 (3)	0.0644 (8)	
H19A	0.4897	0.0873	0.5767	0.077*	
C20	0.3290 (2)	0.1984 (3)	0.6318 (2)	0.0507 (7)	
C21	0.3675 (3)	0.3106 (3)	0.5935 (3)	0.0591 (7)	
H21A	0.2971	0.3793	0.6129	0.071*	
C22	0.4690 (4)	0.2911 (5)	0.6533 (4)	0.0964 (13)	
H22A	0.4458	0.2642	0.7347	0.145*	
H22B	0.4851	0.3691	0.6310	0.145*	
H22C	0.5412	0.2275	0.6327	0.145*	
C23	0.4011 (4)	0.3531 (4)	0.4659 (3)	0.0795 (10)	
H23A	0.3339	0.3678	0.4290	0.119*	
H23B	0.4715	0.2887	0.4439	0.119*	
H23C	0.4195	0.4299	0.4432	0.119*	
C24	0.0521 (3)	0.0961 (3)	0.7572 (3)	0.0713 (9)	
H24A	0.0083	0.1706	0.7832	0.086*	
C25	0.0419 (5)	−0.0170 (5)	0.8554 (4)	0.1118 (16)	
H25A	0.0805	−0.0249	0.9165	0.168*	
H25B	0.0815	−0.0913	0.8314	0.168*	
H25C	−0.0427	−0.0080	0.8817	0.168*	
C26	−0.0091 (4)	0.1096 (5)	0.6632 (4)	0.1061 (15)	
H26A	−0.0044	0.1840	0.6008	0.159*	
H26B	−0.0933	0.1170	0.6908	0.159*	
H26C	0.0308	0.0366	0.6374	0.159*	
Cr1	0.00936 (3)	0.44741 (4)	0.60558 (3)	0.03797 (15)	
H1	0.085 (3)	0.427 (3)	0.480 (3)	0.061 (8)*	
N1	−0.06604 (17)	0.48141 (19)	0.75400 (17)	0.0401 (5)	
N2	0.12678 (17)	0.30968 (19)	0.70837 (17)	0.0420 (5)	
Li1	0.1189 (4)	0.4715 (5)	0.7480 (4)	0.0572 (12)	
O1	0.2231 (2)	0.5599 (2)	0.7256 (2)	0.0769 (7)	
C27A	0.2516 (7)	0.4269 (7)	0.9263 (7)	0.082 (2)*	0.567 (7)
H27A	0.2918	0.4153	0.9878	0.123*	0.567 (7)
H27B	0.2810	0.3498	0.9071	0.123*	0.567 (7)
H27C	0.1650	0.4491	0.9498	0.123*	0.567 (7)
C28A	0.2781 (8)	0.5312 (8)	0.8233 (6)	0.089 (2)*	0.567 (7)
H28A	0.2512	0.6072	0.8452	0.107*	0.567 (7)
H28B	0.3659	0.5081	0.8019	0.107*	0.567 (7)
C29A	0.2652 (9)	0.6611 (7)	0.6526 (7)	0.120 (3)*	0.567 (7)
H29A	0.3536	0.6373	0.6399	0.144*	0.567 (7)
H29B	0.2290	0.7358	0.6774	0.144*	0.567 (7)
C30A	0.2125 (7)	0.6730 (7)	0.5527 (6)	0.081 (2)*	0.567 (7)
H30A	0.2340	0.7365	0.4864	0.122*	0.567 (7)
H30B	0.1251	0.6969	0.5699	0.122*	0.567 (7)
H30C	0.2445	0.5934	0.5377	0.122*	0.567 (7)
C27B	0.2760 (7)	0.4731 (9)	0.8854 (8)	0.061 (2)*	0.433 (7)
H27D	0.3228	0.4577	0.9434	0.091*	0.433 (7)
H27E	0.2972	0.3966	0.8670	0.091*	0.433 (7)
H27F	0.1905	0.5003	0.9132	0.091*	0.433 (7)
C28B	0.3035 (11)	0.5739 (11)	0.7802 (10)	0.117 (4)*	0.433 (7)
H28C	0.2767	0.6562	0.7907	0.140*	0.433 (7)
H28D	0.3879	0.5508	0.7453	0.140*	0.433 (7)
C29B	0.2524 (10)	0.6262 (7)	0.6090 (5)	0.087 (3)*	0.433 (7)
H29C	0.2164	0.6034	0.5634	0.104*	0.433 (7)
H29D	0.3403	0.5925	0.5904	0.104*	0.433 (7)
C30B	0.2175 (9)	0.7676 (7)	0.5671 (8)	0.088 (3)*	0.433 (7)
H30D	0.2386	0.7956	0.4855	0.132*	0.433 (7)
H30E	0.2604	0.7932	0.6029	0.132*	0.433 (7)
H30F	0.1310	0.8045	0.5862	0.132*	0.433 (7)
C31	0.6860 (16)	0.9785 (17)	−0.0580 (15)	0.087 (5)*	0.25
H31A	0.7700	0.9694	−0.0621	0.131*	0.25
H31B	0.6481	1.0550	−0.1151	0.131*	0.25
H31C	0.6818	0.9074	−0.0714	0.131*	0.25
C32	0.6249 (15)	0.9841 (18)	0.0511 (14)	0.091 (5)*	0.25
H32A	0.6315	1.0544	0.0654	0.110*	0.25
H32B	0.6637	0.9070	0.1087	0.110*	0.25
C33	0.4968 (17)	1.000 (2)	0.061 (2)	0.124 (7)*	0.25
H33A	0.4543	1.0899	0.0441	0.149*	0.25
H33B	0.4732	0.9627	0.1422	0.149*	0.25
C34	0.4396 (16)	0.957 (2)	0.002 (2)	0.118 (7)*	0.25
H34A	0.4755	0.9725	−0.0777	0.141*	0.25
H34B	0.4558	0.8664	0.0364	0.141*	0.25
C35	0.3119 (14)	1.0198 (16)	0.0078 (17)	0.077 (4)*	0.25
H35A	0.2774	0.9896	−0.0317	0.116*	0.25
H35B	0.2956	1.1092	−0.0274	0.116*	0.25
H35C	0.2760	1.0033	0.0866	0.116*	0.25

### Atomic displacement parameters (Å^2^)

**Table:**

	*U*^11^	*U*^22^	*U*^33^	*U*^12^	*U*^13^	*U*^23^
C1	0.0394 (13)	0.0572 (15)	0.0428 (14)	−0.0133 (12)	−0.0062 (11)	−0.0232 (12)
C2	0.0393 (14)	0.0529 (15)	0.0470 (14)	−0.0097 (11)	−0.0116 (11)	−0.0202 (12)
C3	0.0317 (12)	0.0545 (15)	0.0403 (13)	−0.0096 (11)	−0.0024 (10)	−0.0238 (11)
C4	0.0412 (14)	0.0521 (15)	0.0544 (16)	−0.0116 (12)	−0.0001 (12)	−0.0265 (13)
C5	0.0483 (16)	0.0519 (16)	0.0649 (18)	−0.0064 (13)	−0.0013 (14)	−0.0255 (14)
C6	0.0354 (14)	0.0658 (19)	0.0613 (18)	−0.0027 (13)	−0.0018 (12)	−0.0253 (15)
C7	0.0344 (14)	0.0680 (18)	0.0529 (16)	−0.0144 (13)	−0.0002 (11)	−0.0215 (14)
C8	0.0357 (13)	0.0540 (15)	0.0436 (13)	−0.0126 (11)	−0.0043 (10)	−0.0205 (12)
C9	0.0422 (15)	0.0575 (16)	0.0609 (17)	−0.0195 (12)	−0.0041 (12)	−0.0231 (14)
C10	0.150 (4)	0.064 (2)	0.076 (3)	−0.037 (3)	0.009 (3)	−0.013 (2)
C11	0.097 (3)	0.079 (2)	0.126 (4)	−0.028 (2)	−0.044 (3)	−0.040 (2)
C12	0.0529 (18)	0.0580 (18)	0.089 (2)	−0.0206 (14)	0.0121 (16)	−0.0402 (17)
C13	0.074 (3)	0.121 (3)	0.135 (4)	−0.048 (2)	0.006 (2)	−0.080 (3)
C14	0.090 (3)	0.062 (2)	0.136 (4)	−0.031 (2)	0.024 (3)	−0.027 (2)
C15	0.0384 (14)	0.0458 (14)	0.0509 (15)	−0.0014 (11)	−0.0114 (11)	−0.0223 (12)
C16	0.0541 (17)	0.0524 (16)	0.0704 (19)	−0.0100 (13)	−0.0069 (14)	−0.0296 (15)
C17	0.078 (2)	0.0478 (17)	0.085 (2)	−0.0088 (16)	−0.0058 (19)	−0.0300 (17)
C18	0.066 (2)	0.0559 (19)	0.081 (2)	0.0078 (16)	−0.0080 (17)	−0.0345 (17)
C19	0.0470 (17)	0.0647 (19)	0.069 (2)	0.0045 (14)	−0.0061 (14)	−0.0319 (16)
C20	0.0390 (14)	0.0561 (16)	0.0529 (16)	−0.0032 (12)	−0.0096 (12)	−0.0234 (13)
C21	0.0391 (15)	0.0716 (19)	0.070 (2)	−0.0142 (13)	0.0008 (13)	−0.0355 (16)
C22	0.091 (3)	0.130 (4)	0.094 (3)	−0.050 (3)	−0.025 (2)	−0.040 (3)
C23	0.079 (3)	0.089 (3)	0.076 (2)	−0.038 (2)	−0.0112 (19)	−0.022 (2)
C24	0.064 (2)	0.0645 (19)	0.101 (3)	−0.0257 (16)	−0.0001 (18)	−0.044 (2)
C25	0.104 (4)	0.141 (4)	0.094 (3)	−0.062 (3)	0.005 (3)	−0.029 (3)
C26	0.076 (3)	0.126 (4)	0.122 (4)	−0.041 (3)	−0.022 (3)	−0.031 (3)
Cr1	0.0292 (2)	0.0452 (2)	0.0423 (2)	−0.00587 (15)	−0.00548 (15)	−0.02277 (17)
N1	0.0296 (10)	0.0497 (12)	0.0450 (12)	−0.0095 (9)	−0.0030 (8)	−0.0239 (10)
N2	0.0338 (11)	0.0467 (11)	0.0477 (12)	−0.0059 (9)	−0.0073 (9)	−0.0235 (10)
Li1	0.049 (3)	0.074 (3)	0.063 (3)	−0.024 (2)	−0.010 (2)	−0.031 (3)
O1	0.0555 (13)	0.0807 (15)	0.1092 (19)	−0.0318 (12)	0.0033 (12)	−0.0445 (14)

### Geometric parameters (Å, °)

**Table:**

C1—C2	1.344 (4)	C24—H24A	1.0000
C1—N1	1.402 (3)	C25—H25A	0.9800
C1—Li1	2.159 (6)	C25—H25B	0.9800
C1—H1A	1.0000	C25—H25C	0.9800
C2—N2	1.396 (3)	C26—H26A	0.9800
C2—Li1	2.159 (6)	C26—H26B	0.9800
C2—H2A	1.0000	C26—H26C	0.9800
C3—C8	1.407 (4)	Cr1—N2	2.023 (2)
C3—C4	1.410 (4)	Cr1—N1	2.030 (2)
C3—N1	1.429 (3)	Cr1—Cr1^i^	2.5780 (8)
C4—C5	1.397 (4)	Cr1—Li1	2.704 (4)
C4—C12	1.510 (4)	Cr1—H1	1.72 (3)
C5—C6	1.373 (4)	N1—Li1	2.204 (5)
C5—H5A	0.9500	N2—Li1	2.206 (5)
C6—C7	1.367 (4)	Li1—O1	1.891 (5)
C6—H6A	0.9500	O1—C28B	1.445 (5)
C7—C8	1.402 (4)	O1—C29A	1.445 (5)
C7—H7A	0.9500	O1—C28A	1.449 (5)
C8—C9	1.517 (4)	O1—C29B	1.450 (5)
C9—C10	1.517 (5)	C27A—C28A	1.535 (6)
C9—C11	1.520 (4)	C27A—H27A	0.9800
C9—H9A	1.0000	C27A—H27B	0.9800
C10—H10A	0.9800	C27A—H27C	0.9800
C10—H10B	0.9800	C28A—H28A	0.9900
C10—H10C	0.9800	C28A—H28B	0.9900
C11—H11A	0.9800	C29A—C30A	1.527 (6)
C11—H11B	0.9800	C29A—H29A	0.9900
C11—H11C	0.9800	C29A—H29B	0.9900
C12—C14	1.518 (5)	C30A—H30A	0.9800
C12—C13	1.525 (5)	C30A—H30B	0.9800
C12—H12A	1.0000	C30A—H30C	0.9800
C13—H13A	0.9800	C27B—C28B	1.528 (7)
C13—H13B	0.9800	C27B—H27D	0.9800
C13—H13C	0.9800	C27B—H27E	0.9800
C14—H14A	0.9800	C27B—H27F	0.9800
C14—H14B	0.9800	C28B—H28C	0.9900
C14—H14C	0.9800	C28B—H28D	0.9900
C15—C20	1.402 (4)	C29B—C30B	1.531 (6)
C15—C16	1.405 (4)	C29B—H29C	0.9900
C15—N2	1.438 (3)	C29B—H29D	0.9900
C16—C17	1.398 (4)	C30B—H30D	0.9800
C16—C24	1.524 (5)	C30B—H30E	0.9800
C17—C18	1.376 (3)	C30B—H30F	0.9800
C17—H17A	0.9500	C31—C32	1.433 (16)
C18—C19	1.372 (3)	C31—H31A	0.9800
C18—H18A	0.9500	C31—H31B	0.9800
C19—C20	1.398 (4)	C31—H31C	0.9800
C19—H19A	0.9500	C32—C33	1.482 (17)
C20—C21	1.505 (4)	C32—H32A	0.9900
C21—C22	1.526 (5)	C32—H32B	0.9900
C21—C23	1.531 (5)	C33—C34	1.496 (17)
C21—H21A	1.0000	C33—H33A	0.9900
C22—H22A	0.9800	C33—H33B	0.9900
C22—H22B	0.9800	C34—C35	1.444 (16)
C22—H22C	0.9800	C34—H34A	0.9900
C23—H23A	0.9800	C34—H34B	0.9900
C23—H23B	0.9800	C35—H35A	0.9800
C23—H23C	0.9800	C35—H35B	0.9800
C24—C26	1.515 (6)	C35—H35C	0.9800
C24—C25	1.517 (6)		
			
C2—C1—N1	116.1 (2)	N1—Cr1—Cr1^i^	139.45 (6)
C2—C1—Li1	71.9 (2)	N2—Cr1—Li1	53.31 (13)
N1—C1—Li1	73.0 (2)	N1—Cr1—Li1	53.20 (12)
C2—C1—H1A	121.9	Cr1^i^—Cr1—Li1	133.28 (12)
N1—C1—H1A	121.9	N2—Cr1—H1	99.1 (10)
Li1—C1—H1A	121.9	N1—Cr1—H1	173.4 (10)
C1—C2—N2	116.8 (2)	Cr1^i^—Cr1—H1	41.0 (10)
C1—C2—Li1	71.9 (2)	Li1—Cr1—H1	120.8 (10)
N2—C2—Li1	73.20 (19)	C1—N1—C3	116.5 (2)
C1—C2—H2A	121.6	C1—N1—Cr1	113.00 (15)
N2—C2—H2A	121.6	C3—N1—Cr1	124.17 (15)
Li1—C2—H2A	121.6	C1—N1—Li1	69.5 (2)
C8—C3—C4	119.8 (2)	C3—N1—Li1	141.9 (2)
C8—C3—N1	119.9 (2)	Cr1—N1—Li1	79.28 (14)
C4—C3—N1	120.3 (2)	C2—N2—C15	115.3 (2)
C5—C4—C3	118.6 (3)	C2—N2—Cr1	113.15 (15)
C5—C4—C12	120.4 (3)	C15—N2—Cr1	125.94 (16)
C3—C4—C12	120.9 (2)	C2—N2—Li1	69.51 (19)
C6—C5—C4	121.8 (3)	C15—N2—Li1	139.9 (2)
C6—C5—H5A	119.1	Cr1—N2—Li1	79.38 (15)
C4—C5—H5A	119.1	O1—Li1—C2	147.1 (3)
C7—C6—C5	119.3 (3)	O1—Li1—C1	153.6 (3)
C7—C6—H6A	120.3	C2—Li1—C1	36.26 (13)
C5—C6—H6A	120.3	O1—Li1—N1	146.7 (3)
C6—C7—C8	121.8 (3)	C2—Li1—N1	64.56 (16)
C6—C7—H7A	119.1	C1—Li1—N1	37.46 (12)
C8—C7—H7A	119.1	O1—Li1—N2	136.9 (3)
C7—C8—C3	118.6 (2)	C2—Li1—N2	37.29 (12)
C7—C8—C9	118.8 (2)	C1—Li1—N2	64.60 (16)
C3—C8—C9	122.6 (2)	N1—Li1—N2	71.92 (16)
C10—C9—C8	112.3 (3)	O1—Li1—Cr1	132.7 (3)
C10—C9—C11	110.1 (3)	C2—Li1—Cr1	71.45 (14)
C8—C9—C11	112.2 (3)	C1—Li1—Cr1	71.71 (14)
C10—C9—H9A	107.3	N1—Li1—Cr1	47.53 (10)
C8—C9—H9A	107.3	N2—Li1—Cr1	47.31 (10)
C11—C9—H9A	107.3	C28B—O1—C29A	70.4 (6)
C9—C10—H10A	109.5	C29A—O1—C28A	98.4 (5)
C9—C10—H10B	109.5	C28B—O1—C29B	101.9 (7)
H10A—C10—H10B	109.5	C28A—O1—C29B	130.0 (6)
C9—C10—H10C	109.5	C28B—O1—Li1	143.1 (5)
H10A—C10—H10C	109.5	C29A—O1—Li1	146.2 (4)
H10B—C10—H10C	109.5	C28A—O1—Li1	114.7 (4)
C9—C11—H11A	109.5	C29B—O1—Li1	114.1 (5)
C9—C11—H11B	109.5	C28A—C27A—H27A	109.5
H11A—C11—H11B	109.5	C28A—C27A—H27B	109.5
C9—C11—H11C	109.5	H27A—C27A—H27B	109.5
H11A—C11—H11C	109.5	C28A—C27A—H27C	109.5
H11B—C11—H11C	109.5	H27A—C27A—H27C	109.5
C4—C12—C14	114.3 (3)	H27B—C27A—H27C	109.5
C4—C12—C13	109.5 (3)	O1—C28A—C27A	115.7 (6)
C14—C12—C13	111.8 (3)	O1—C28A—H28A	108.4
C4—C12—H12A	107.0	C27A—C28A—H28A	108.4
C14—C12—H12A	107.0	O1—C28A—H28B	108.4
C13—C12—H12A	107.0	C27A—C28A—H28B	108.4
C12—C13—H13A	109.5	H28A—C28A—H28B	107.4
C12—C13—H13B	109.5	O1—C29A—C30A	92.9 (5)
H13A—C13—H13B	109.5	O1—C29A—H29A	113.1
C12—C13—H13C	109.5	C30A—C29A—H29A	113.1
H13A—C13—H13C	109.5	O1—C29A—H29B	113.1
H13B—C13—H13C	109.5	C30A—C29A—H29B	113.1
C12—C14—H14A	109.5	H29A—C29A—H29B	110.5
C12—C14—H14B	109.5	C29A—C30A—H30A	109.5
H14A—C14—H14B	109.5	C29A—C30A—H30B	109.5
C12—C14—H14C	109.5	H30A—C30A—H30B	109.5
H14A—C14—H14C	109.5	C29A—C30A—H30C	109.5
H14B—C14—H14C	109.5	H30A—C30A—H30C	109.5
C20—C15—C16	120.0 (2)	H30B—C30A—H30C	109.5
C20—C15—N2	120.7 (2)	C28B—C27B—H27D	109.5
C16—C15—N2	119.3 (2)	C28B—C27B—H27E	109.5
C17—C16—C15	118.8 (3)	H27D—C27B—H27E	109.5
C17—C16—C24	119.0 (3)	C28B—C27B—H27F	109.5
C15—C16—C24	122.1 (2)	H27D—C27B—H27F	109.5
C18—C17—C16	121.2 (3)	H27E—C27B—H27F	109.5
C18—C17—H17A	119.4	O1—C28B—C27B	88.8 (6)
C16—C17—H17A	119.4	O1—C28B—H28C	113.8
C19—C18—C17	119.6 (3)	C27B—C28B—H28C	113.8
C19—C18—H18A	120.2	O1—C28B—H28D	113.8
C17—C18—H18A	120.2	C27B—C28B—H28D	113.8
C18—C19—C20	121.4 (3)	H28C—C28B—H28D	111.1
C18—C19—H19A	119.3	O1—C29B—C30B	121.4 (7)
C20—C19—H19A	119.3	O1—C29B—H29C	107.0
C19—C20—C15	118.9 (3)	C30B—C29B—H29C	107.0
C19—C20—C21	118.7 (3)	O1—C29B—H29D	107.0
C15—C20—C21	122.5 (2)	C30B—C29B—H29D	107.0
C20—C21—C22	112.8 (3)	H29C—C29B—H29D	106.7
C20—C21—C23	111.6 (3)	C29B—C30B—H30D	109.5
C22—C21—C23	110.7 (3)	C29B—C30B—H30E	109.5
C20—C21—H21A	107.2	H30D—C30B—H30E	109.5
C22—C21—H21A	107.2	C29B—C30B—H30F	109.5
C23—C21—H21A	107.2	H30D—C30B—H30F	109.5
C21—C22—H22A	109.5	H30E—C30B—H30F	109.5
C21—C22—H22B	109.5	C32—C31—H31A	109.5
H22A—C22—H22B	109.5	C32—C31—H31B	109.5
C21—C22—H22C	109.5	H31A—C31—H31B	109.5
H22A—C22—H22C	109.5	C32—C31—H31C	109.5
H22B—C22—H22C	109.5	H31A—C31—H31C	109.5
C21—C23—H23A	109.5	H31B—C31—H31C	109.5
C21—C23—H23B	109.5	C31—C32—C33	111.6 (17)
H23A—C23—H23B	109.5	C31—C32—H32A	109.3
C21—C23—H23C	109.5	C33—C32—H32A	109.3
H23A—C23—H23C	109.5	C31—C32—H32B	109.3
H23B—C23—H23C	109.5	C33—C32—H32B	109.3
C26—C24—C25	109.6 (4)	H32A—C32—H32B	108.0
C26—C24—C16	110.0 (3)	C32—C33—C34	128.5 (18)
C25—C24—C16	113.8 (3)	C32—C33—H33A	105.2
C26—C24—H24A	107.7	C34—C33—H33A	105.2
C25—C24—H24A	107.7	C32—C33—H33B	105.2
C16—C24—H24A	107.7	C34—C33—H33B	105.2
C24—C25—H25A	109.5	H33A—C33—H33B	105.9
C24—C25—H25B	109.5	C35—C34—C33	111.6 (16)
H25A—C25—H25B	109.5	C35—C34—H34A	109.3
C24—C25—H25C	109.5	C33—C34—H34A	109.3
H25A—C25—H25C	109.5	C35—C34—H34B	109.3
H25B—C25—H25C	109.5	C33—C34—H34B	109.3
C24—C26—H26A	109.5	H34A—C34—H34B	108.0
C24—C26—H26B	109.5	C34—C35—H35A	109.5
H26A—C26—H26B	109.5	C34—C35—H35B	109.5
C24—C26—H26C	109.5	H35A—C35—H35B	109.5
H26A—C26—H26C	109.5	C34—C35—H35C	109.5
H26B—C26—H26C	109.5	H35A—C35—H35C	109.5
N2—Cr1—N1	79.44 (8)	H35B—C35—H35C	109.5
N2—Cr1—Cr1^i^	139.85 (6)		

Symmetry codes: (i) −*x*, −*y*+1, −*z*+1.
